# Habitat productivity constrains the distribution of social spiders across continents – case study of the genus *Stegodyphus*

**DOI:** 10.1186/1742-9994-10-9

**Published:** 2013-02-23

**Authors:** Marija Majer, Jens-Christian Svenning, Trine Bilde

**Affiliations:** 1 Department of Bioscience, Aarhus University, Ny Munkegade 116, Aarhus-C, 8000, Denmark

**Keywords:** Macroecology, Social spiders, Group living, Habitat productivity, Prey availability, Insect biomass

## Abstract

**Introduction:**

Sociality has evolved independently multiple times across the spider phylogeny, and despite wide taxonomic and geographical breadth the social species are characterized by a common geographical constrain to tropical and subtropical areas. Here we investigate the environmental factors that drive macro-ecological patterns in social and solitary species in a genus that shows a Mediterranean–Afro-Oriental distribution (*Stegodyphus*). Both selected drivers (productivity and seasonality) may affect the abundance of potential prey insects, but seasonality may further directly affect survival due to mortality caused by extreme climatic events. Based on a comprehensive dataset including information about the distribution of three independently derived social species and 13 solitary congeners we tested the hypotheses that the distribution of social *Stegodyphus* species relative to solitary congeners is: (1) restricted to habitats of high vegetation productivity and (2) constrained to areas with a stable climate (low precipitation seasonality).

**Results:**

Using spatial logistic regression modelling and information-theoretic model selection, we show that social species occur at higher vegetation productivity than solitary, while precipitation seasonality received limited support as a predictor of social spider occurrence. An analysis of insect biomass data across the *Stegodyphus* distribution range confirmed that vegetation productivity is positively correlated to potential insect prey biomass.

**Conclusions:**

Habitat productivity constrains the distribution of social spiders across continents compared to their solitary congeners, with group-living in spiders being restricted to areas with relatively high vegetation productivity and insect prey biomass. As known for other taxa, permanent sociality likely evolves in response to high predation pressure and imposes within-group competition for resources. Our results suggest that group living is contingent upon productive environmental conditions where elevated prey abundance meet the increased demand for food of social groups.

## Introduction

Social interactions in animals may range from communal foraging and predator defence to highly complex cooperative societies with morphological and behavioural differentiation among group members. The evolution of sociality is intriguing as the benefits of group living are offset by costs of competition over resources and reproduction, and the cost benefit ratio is shaped by ecological conditions and genetic relationships. Groups may form in response to environmental variables such as food or predators, or in response to ecological constraints if individuals are unable to survive and reproduce solitarily. Understanding the relationship between ecological conditions and social behaviour therefore provides insight to environmental conditions under which group living evolves.

Social spiders live in groups ranging from hundreds to thousands of individuals, where females cooperate in prey capture, web maintenance, predator defence and brood care [1,2]. Permanent sociality in spiders is rare, with fewer than 25 known social species of more than 43.000 known spider species [3]. Interestingly, there are at least 18 independent origins of sociality [4], suggesting that sociality may evolve in response to common environmental conditions. Indeed, social spiders are confined to tropical and subtropical regions [2,5]. Phylogenetic analyses suggest that sociality is derived from solitary ancestors and the social clades appear to undergo little diversification once sociality has evolved [4,6]. These patterns are in stark contrast to the social insects, which are distributed worldwide and were social clades have diversified highly successfully [7]. Social spiders show convergent evolution of a number of traits – denoted as the social syndrome [8] - these include the loss of juvenile dispersal, regular inbreeding, female-biased primary sex ratio, and post mating dispersal where mated females initiate new colonies [8,9]. The restricted geographical distribution and multiple independent origins of sociality suggest that sociality in spiders is contingent upon certain consistent environmental conditions that favour the evolution of similar life history traits. Our aim in the present study was to improve our understanding of the conditions that correlate with permanent sociality in a genus that includes social and solitary species with a Mediterranean–Afro-Oriental distribution (*Stegodyphus* spp.).

Within arthropod lineages, inter- and intraspecific variation in the degree of social behaviour may coincide with gradients in environmental factors such as temperature and precipitation [10,11]. For example, bees show more complex societies in lower latitudes, while less social forms are abundant in the more species-rich (and xeric) subtropics [12]. Halictine bees show a similar pattern where the degree of sociality increases and becomes more complex with decreasing latitude [11,13]. Ants show a different tendency as colony sizes increase with increasing latitude [14]. In contrast, termites do not show consistent patterns of variation in colony size with geographical gradients [15]. Social spiders appear to exhibit a gradient where social species are found mainly in the tropics or subtropics while their solitary congeners extend well into temperate regions. In the New World genus *Anelosimus*, several social species are found only in the lowlands of South America indicating a gradient where sociality decreases with increasing altitude (I. Agnarsson *pers. obs*.) – similar to the latitudinal gradient of social spiders as a whole.

Several related hypotheses for the tropical and subtropical distribution of social spiders propose that sociality is contingent on high habitat productivity. One of the main constraints on group living is the elevated demand for food. Higher plant productivity of the wetter part of the tropics should cascade into production of more and larger potential prey [16,17] that meets the food demands of social spiders [18,19]. Another feature associated with these areas is relatively low seasonality that results in longer growing seasons which should result in higher insect prey productivity [11] and thus a continuous supply of food [20]. This facilitates delayed dispersal from the maternal nest which is one of the key elements in the formation of groups [8,21,22]. Furthermore, low seasonality could promote group living by facilitating continuous communal foraging and brood care [11,23]. Finally, it is possible that habitats with lower climatic seasonality are generally environmentally more benign because of fewer climatic extremes, e.g. temperature and rainfall fluctuations. Social spiders are highly inbred and show characteristic bust-and-boom colony dynamics where entire populations go extinct seemingly in response to some environmental hazard [2]. Hence, they may be particularly sensitive to climatic fluctuations. Highly synchronised populations may also be subject to high rates of extinction, for example due to parasite attacks, as shown for populations of halictine [24] and carpenter bees [25].

We examined these hypotheses in the spider genus *Stegodyphus* (Eresidae), which includes three independently evolved social species and 18+ solitary species [26,27]. *Stegodyphus* species occur in dry savannah-like areas across the African continent, around the Mediterranean basin and in the Middle East and South-East Asia [3,27,28]. We tested whether the social species occur in habitats with higher productivity compared to their solitary congeners [18,29]. We further examined whether the social species occur under more constant year-round climatic conditions [7,30]; by examining whether social species occur in regions with lower precipitation seasonality compared with their solitary congeners. To test these hypotheses, we performed logistic regression on presences of social vs. presences of solitary species with environmental layers in 1-km^2^ resolution. To support the functional basis for our modelling results, we also assessed whether insect biomass co-varies with productivity and/or precipitation seasonality across the *Stegodyphus* distribution ranges.

## Results

**Figure 1:**
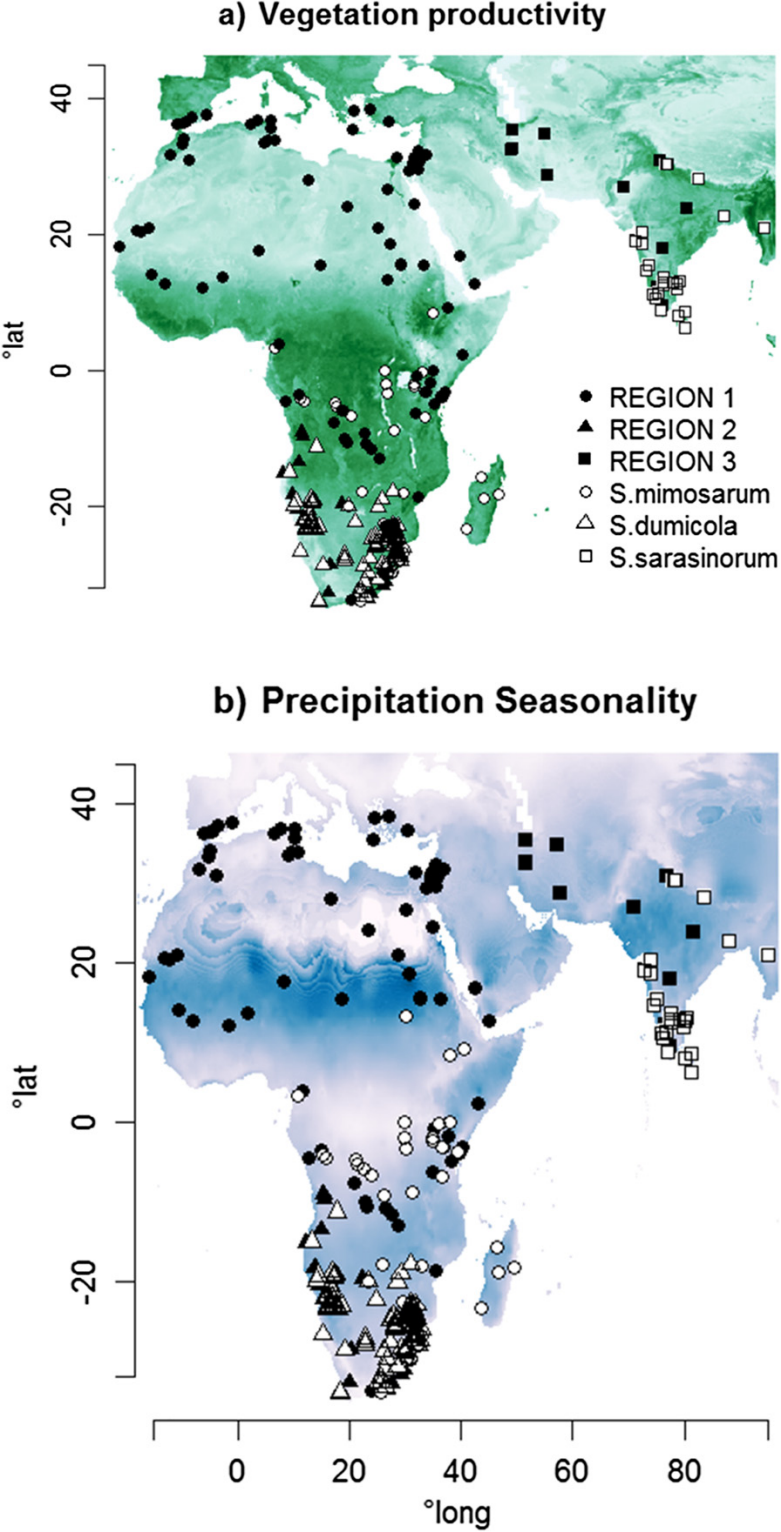
**Distribution map of *Stegodyphus *spp. on a continental gradient of two environmental predictors, (a) vegetation productivity (unitless ratio) and (b) precipitation seasonality (coefficient of variation of monthly means, mm) (b).** Three regions, defined to separate the distributions of the social species, are indicated by circles (region 1), triangles (region 2) and rectangles (region 3). Empty and filled symbols indicate the occurrences of social and solitary species, respectively (n = 366 occurrences in total). For inserts of species maps in the South African region where the spider distribution records are very dense, see Additional file 1: Figure S1. The darker the green in (**a**), the more productive the continental area is. Likewise, the bluer the continental area in (**b**), the more seasonal it is in precipitation patterns.

Our inventory of *Stegodyphus* occurrence records confirmed the broad distribution of the genus across Africa, southern Mediterranean, Middle East, and southern Asia (Figure 1). The northern-most distributed species is *S. lineatus* (70.20; 46.033); southernmost *S. tentoriicola* (23.89; -33.84); easternmost *S. sarasinorum* (95.00; 21.00) and westernmost is *S. manicatus* (−17.44; 14.67). These occurrence data furthermore confirmed that the three social species are restricted to more tropical environments than the genus as whole (Figure 1). As shown in Figure 1a, all species (solitary and social) in this genus occur in warm and dry areas, while they are absent from forest habitats, like all other species in the family Eresidae.

**Figure 2:**
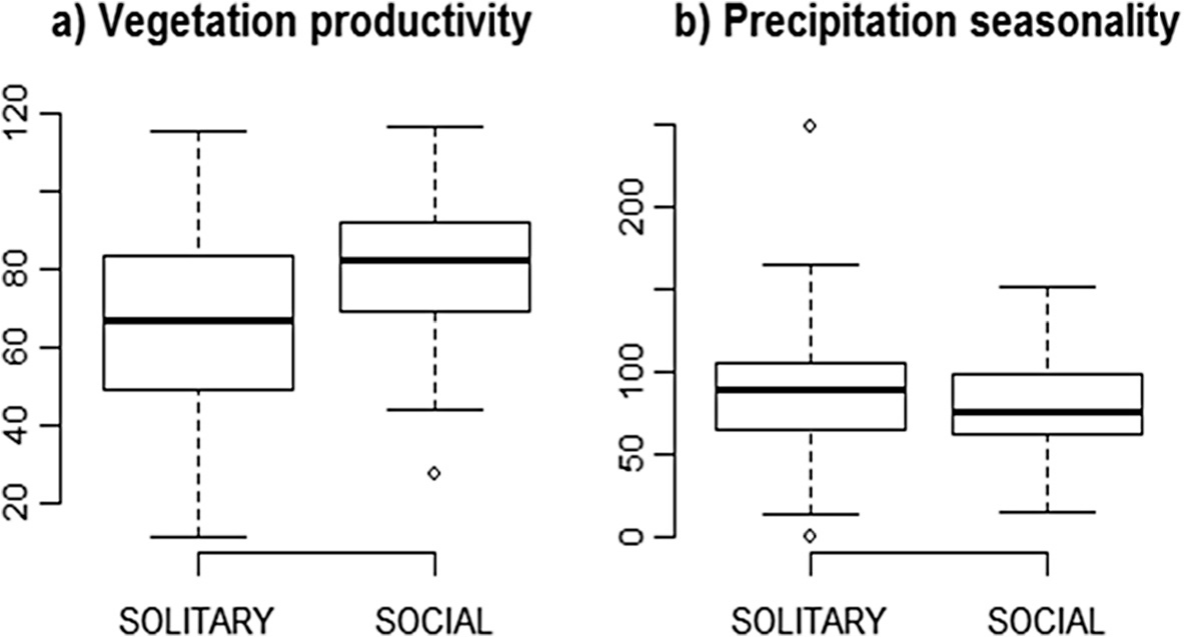
**Boxplots of (a) vegetation productivity and (b) precipitation seasonality for occurrences of social and solitary *Stegodyphus *species (n = 193 and 173, respectively).** The extremes, the inter-quartile range, and the median are shown.

The univariate Wilcoxon sum rank tests showed that social species occur in areas of relatively high productivity (W = 10735.5, p < 0.0001; n = 197 for social species and 169 for solitary species respectively, Figure 2a), and we also found some support for the occurrence of social species in areas of relatively low precipitation seasonality (W = 19505.5, p < 0.05; n = 193 for social species and 173 for solitary species, Figure 2b; but see also Additional file 1: Figure S2).

**Figure 3:**
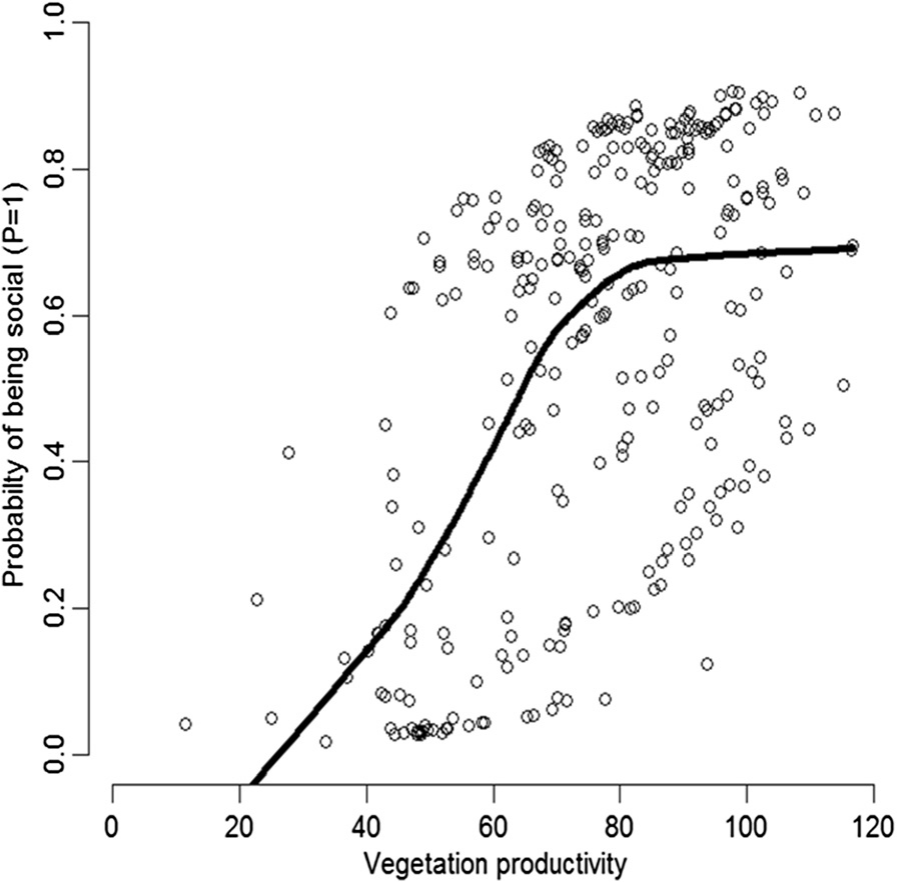
**Probability that a *Stegodyphus *occurrence records belongs to social species as a function of vegetation productivity.** The probability function was estimated by a logistic regression with vegetation productivity and spatial filters as predictors (Table 1). The line indicates a response curve estimated by smoothing the probabilities predicted by the vegetation productivity + spatial filters model.

In the logistic regression modelling, model selection provided strong support for a positive effect of vegetation productivity on the occurrence of social relative to solitary *Stegodyphus* species (Tables 1, 2; Figure 3). Neither precipitation seasonality nor the regional effect and its interactions were significant drivers of social *Stegodyphus* species occurrence (Tables 1, 2). The weak region effect indicates that the three social species exhibited consistent environmental relationships within the different study regions. The logistic regression models explained between 36-42% of social species presence-absence (Table 1), with the mean TSS score across models 0.320 ± 0.006, i.e., indicating fair predictive ability of social vs. solitary *Stegodyphus* occurrences. There was no residual spatial autocorrelation in the model residuals (non-significant Moran’s I; Additional file 1: Figure S3).

**Table 1:** **Determinants that a given *Stegodyphus *occurrence record belongs to a social species, assessed by logistic regression modelling with information-theoretic model selection**

**Model/predictors**						**K**	**∆ AIC**	**w**_***i***_	**TSS**	**R**^**2**^
**Region**			**GVI**		**SF**	**9**	**0.321**	**0.271**	**0.303**	**0.425**
**Region**			**GVI**	I (Region* GVI)	**SF**	**10**	**2.069**	**0.113**	**0.318**	**0.425**
**Region**	**PSea**		**GVI**		**SF**	**10**	**1.965**	**0.119**	**0.308**	**0.425**
**Region**	PSea	I (Region* PSea)	GVI	I (Region* GVI)	SF	12	5.260	0.023	0.324	0.427
**Region**	PSea				SF	9	20.886	0.000	0.328	0.371
**Region**	PSea	I (Region* PSea)			SF	10	22.727	0.000	0.306	0.372
	**PSea**		**GVI**		**SF**	**8**	**1.406**	**0.157**	**0.305**	**0.417**
			**GVI**		**SF**	**7**	**0.000**	**0.318**	**0.306**	**0.415**
	PSea				SF	7	18.428	0.000	0.342	0.368
					SF	6	19.752	0.000	0.355	0.359

Abbreviations of the predictors used in each of the 10 models for logit link, are as follows (without the intercept): Region for regional variable (combination of 2 binaries); GVI for vegetation productivity; PSea for precipitation seasonality; I (Region* GVI and Region*PSea) for interaction terms; and SF for six spatial filters used. Best supported models are shown in bold. K is the number of model parameters, ∆ AIC are AIC differences, w*i* Akaike weights of each model; and TSS is the true skill statistics score of each model (see Methods section for more explanation on the last three).

**Table 2:** **Akaike weights of the models including each of the non-spatial predictors in logistic regression on presence/absence of social *Stegodyphus *spp**

**Variable**	**β**_**A**_	**OR**_**soc**_	**P**_**soc**_	**w**_***i***_
**GVI**	1.366	3.921	0.981	1.000
**PSea**	−0.057	0.945	0.720	0.299
**Region**	-	-	-	0.525
**Region × GVI**	-	-	-	0.136
**Region × PSea**	-	-	-	0.023

Akaike weights of all the models (Table 1) were used for calculating the multi-model coefficient estimates, the sign of which are given in the table. The sums of Akaike weights of models containing each of the variables are given (w*i*). Vegetation productivity (GVI, Figure 1a) of the habitats received the most support. Other abbreviations used: precipitation variation (PSea; Figure 1b), two binary regional variables (Region binary 1/2) and the interactions of the two environmental predictors with the former categorical variable for region (Region*GVI, Region*PSea; Figure 1a & b) of each of the three social species. The coefficient estimates for the main regional and interaction effects are not given, as they are difficult to interpret and only weakly supported. β_A_ denotes the model-averaged regression coefficients; OR_soc_ is the odds ratio of being social; and Psoc is the probability of finding a social species with one unit increase of the respective environmental variable.

**Figure 4:**
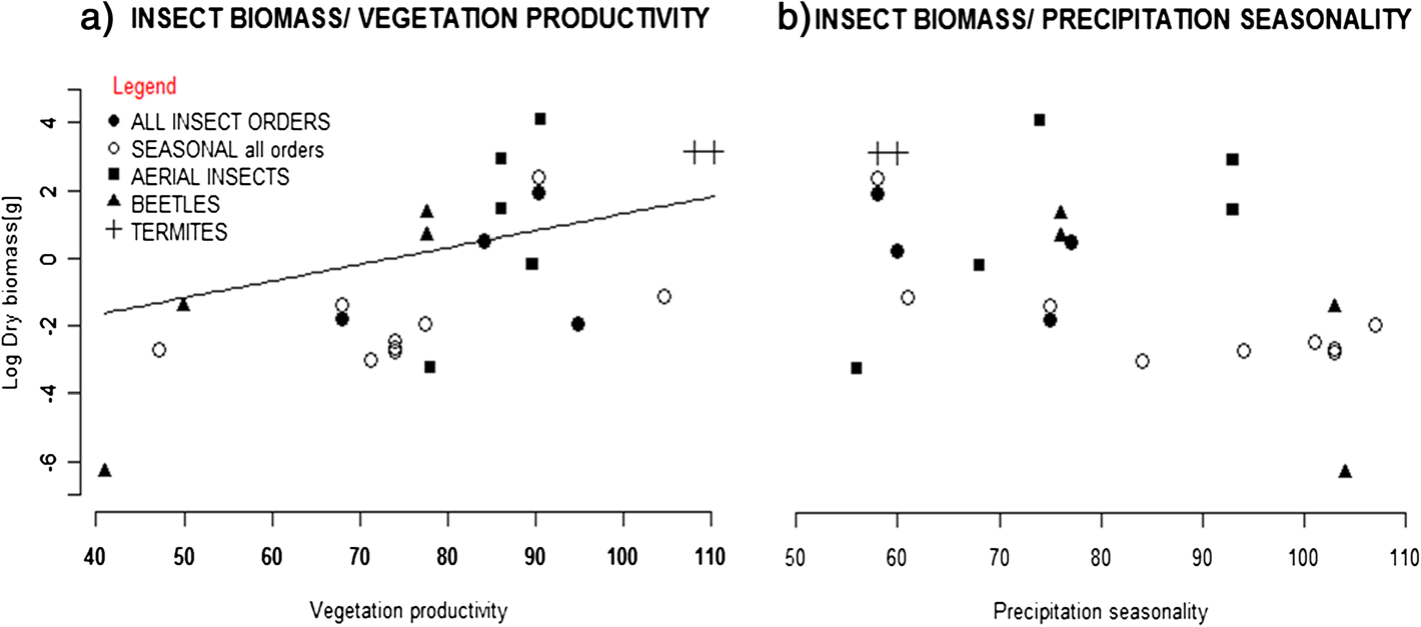
**Insect biomass as a function of (a) vegetation productivity, and of (b) precipitation seasonality.** Annual and seasonal insect biomass estimates from the study region are plotted on the vegetation productivity and precipitation seasonality gradients, with the line in (**a**) showing the significant partial coefficient estimates for vegetation productivity from mixed model effects with log insect biomass as a response (Table 3), with vegetation productivity, precipitation seasonality and taxon as fixed; and sampling effort as a random effect.

Insect biomass increased with increasing vegetation productivity, but was unrelated to precipitation seasonality (Table 3; Figure 4). We obtained similar results, whether or not the biomass estimates were taxon specific or pooled for the relevant time of season.

**Table 3:** Analysis of variance table for linear mixed-effects model on annual and seasonal insect biomass estimates, with vegetation productivity (GVI), precipitation seasonality (PSea), taxon and season as fixed effects, and sampling effort as a random factor (number of observations: 24)

**Variable**	**dF**	**F-value**	**p-value**
**GVI**	**1**	**7.825**	**0.031**
**PSea**	1	2.059	0.201
**Taxon**	3	0.419	0.743
**Season**	1	0.855	0.355

dF is the numerator degrees of freedom in the ANOVA analysis, while the denominator dF is left out since linear models with mixed-effects were used. The significant effect is bolded.

## Discussion

We used spatial modelling to examine two hypotheses for the restriction of group-living *Stegodyphus* spiders to tropical and subtropical regions. By analysing the continental-scale environmental relationships of the genus *Stegodyphus*, we found clear support for the hypothesis that group-living spiders are restricted to habitats with high vegetation productivity relative to solitary congeners. This corroborates our hypothesis that habitats with high vegetation productivity support the high prey abundance that is required to sustain the food requirements of social groups. Notably, we found a strong positive relationship of social *Stegodyphus* species’ occurrence to vegetation productivity and a strong positive relationship between insect biomass and vegetation productivity. These results are consistent with empirical work suggesting that group living in spiders is tightly associated with high prey abundance, whether this applies to insect abundance or size, both of which correlate positively with insect biomass [18,19,29,31]. We note that our results also confirmed that even social *Stegodyphus* spp. are absent from forest environments, just like all other species in the family Eresidae. This absence is thus not evidence for a particular constraint on the distribution of social vs. solitary species within the *Stegodyphus* genus, but rather indicates niche conservatism at the family level.

The multivariate analyses did not support the occurrence of social *Stegodyphus* in areas of low precipitation seasonality compared with their solitary congeners (Tables 1, 2), although an initial comparison of habitats provided weak evidence that social spiders may be restricted to habitats of relatively lower precipitation seasonality (Figure 2 and Additional file 1: Figure S2). We tested the hypothesis that social species might be limited to less variable environments to cope with demands of group living, for example continuous prey supply, as several studies have found correlations between insect abundance and biomass with seasonal rainfall patterns [32,33]. Under low precipitation seasonality prey availability might vary less over the year, which reduces variance in prey capture rates and meets the constant demands of foraging groups. However, we did not find substantial support for this hypothesis in the regression analyses; neither did we find any consistent relationship between insect biomass and precipitation seasonality (Figure 4b). Our analyses therefore strongly indicate that prey availability *per se* rather than continuity in prey supply is the key factor for the formation and maintenance of social *Stegodyphus* groups. Moreover, this is in agreement with *Stegodyphus* spp. occurring in more seasonal habitats (in contrast to *e.g. Anelosimus*), and going dormant over times of the season when prey is particularly scarce. Thus, the quantity of food supply at certain life-stages might be more important than the constancy of prey.

While food and predators are widely recognised as two major factors underlying the formation of groups, the evolution of cooperative breeding is not necessarily facilitated by single factors alone. For example, environmental constraints such as lack of suitable habitat and genetic pre-disposition for helping relatives may act in concert to promote cooperative breeding in birds and social insects [34-36]. Sociality in spiders is favoured by enhanced survival with increasing group size [37], and kin-selected benefits of cooperation [6,38], where the former is likely to result from heavy predation pressure that wipe out small colonies [39]. However, group living is associated with fecundity costs as per capita lifetime reproductive success decreases with increasing group size [38,40]. Hence, group living spiders face a trade-off between survival benefits and reproductive costs. Our analyses show that three independently derived social *Stegodyphus* species with similar life-histories also share similar habitat requirements of high vegetation productivity, suggesting that productivity needs to exceed a certain threshold to tip the balance in favour of group living. Importantly, the negligible region and region-productivity effects suggest that the three social species have consistent vegetation productivity requirements (Table 2; Additional file 1: Figure S2). Only habitats that negate within-group competition which causes fitness decline should therefore be suitable to facilitate the formation and maintenance of groups. The facultative social Neotropical spider *Anelosimus studiosus* shows a life-history pattern consistent with this expectation: females breed solitarily under relaxed ecological conditions (i.e. higher temperature surrounding the nest sites) whereas they engage in cooperative breeding under constrained ecological conditions with increased risk of mortality [41].

In contrast to the patterns discussed above, where environmental constraints favour group living under certain environmental conditions, some animal species form groups in response to resources to exploit them more efficiently, in which case resource availability may be the single factor promoting group formation. This applies to colonial spiders, which differ from permanently-social spiders by sharing a common silk frame, but defend individual capture webs within the group, do not hunt communally, nor show cooperative breeding [8]. The colonial spiders also occur mainly in the tropics and groups form through aggregations around abundant resources [42]. In contrast, the social spiders likely evolved group living through delayed dispersal [2]. While different proximate factors are likely involved in group formation, i.e., foraging benefits for colonial spiders and survival benefits for permanently social spiders, both groups are characterized by a distributional range within the tropics, which corroborate that prey availability in sufficient supply is an important environmental factor facilitating group living [2,43].

Our results provide evidence that the link between the occurrence of social *Stegodyphus* species and vegetation productivity is related to prey availability. Notably, vegetation productivity in the general distribution area for the genus correlated positively with insect biomass. Other studies have likewise shown that the abundance of vegetation and canopy cover influences the amount and seasonal distribution of insects [44,45]. Furthermore, multiple previous studies on arthropod distributions have found that remotely sensed vegetation structure descriptors perform well as predictors of arthropod species occurrences and species richness across large scales [46,47].

Prey size has been hypothesized to constrain the distribution of social spiders, based on field studies showing such a pattern for four American *Anelosimus* species (2 social and 2 subsocial) [19]. Our data suggest that prey numbers are a major driver of *Stegodyphus* distribution patterns. Firstly, *Stegodyphus* species occur in habitats that are generally less diverse than tropical rainforests (habitats of social *Anelosimus* spp.), and we argue that the range of prey species is smaller and may therefore generally span a shorter body-size gradient. Secondly, we show that insect abundance is positively correlated to habitat productivity which is the most important predictor of *Stegodyphus* distribution in our models. Though we cannot rule out that prey body-size may be an important additional factor, we suggest that our approach provides a more general and valid explanation of the observed distribution patterns in social and solitary *Stegodyphus* species based on the relationship between vegetation productivity and prey biomass.

An additional explanation for the importance of vegetation productivity for the occurrence of social *Stegodyphus* species may be their link to vegetation complexity. Group-living *Stegodyphus* require certain structures for establishing colonies and capture webs, and a denser vegetation cover would offer more vertically and structurally diverse micro-niche space. Evidence for this explanation was recently found in the tropical rainforest *Anelosimus* species, where permanently group living species require sturdier vegetation for nest building [48]. Structural complexity of habitats has been shown to provide important environmental cues to web-building in spiders [49], in particular due to their predatory function in the ecosystems [50]. This can also have an effect on species traits in the guild through prey availability [48,51]. Considering these factors, we could expect that social *Stegodyphus* should be found in areas of increasing complexity in vegetation structure - relative to their solitary congeners, respectively.

## Conclusions

Our study suggests that social *Stegodyphus* species have a reduced habitat niche width compared to their solitary congeners. We found a consistent distribution pattern for three independently derived social species in areas of high vegetation productivity, and we show that insect prey biomass is positively correlated to vegetation productivity. These patterns provide empirical support for the hypothesis that group living is contingent upon benign environmental conditions that negate fitness loss resulting from competition for resources within groups. Similar to group formation in many other taxa, permanent sociality and cooperative breeding likely evolve in response to high predation pressure, and our study suggests that group living is only an evolutionarily stable strategy under conditions with high ambient insect prey availability.

## Methods

### Species data

*Stegodyphus* belongs to the family Eresidae, that show extended maternal care [52,53] and matriphagy (spiderlings consume their mother) [52,54,55]. After matriphagy, the juveniles of solitary species disperse out of the maternal nest to live solitarily, while the juveniles of the three social species remain in the maternal nest and cooperate in prey capture and brood care [56]. The social species form colonies in trees or large shrubs with a shared retreat and large capture web complexes [57]. The solitary species are typically found in small shrubs or low vegetation. To compile a data set for species distribution analyses, species records were collected from all available sources (publications, online databases and field records) [27,28,58,59] & references therein. Every *Stegodyphus* record obtained was georeferenced [60] to produce a spatial map of locality records for all species (Figure 1). Several old records from the early 20th century were neglected due to poor locality descriptions [61], and we assured that the produced range maps comply with literature descriptions [28]. The dataset consisted of 366 species records of 16 species in total (Additional file 1: Table S1).

### Environmental variables

We tested the effect of habitat productivity on social spider range using the annual Globalised Vegetation Index (GVI henceforth referred to as vegetation productivity) as predictor variable. GVI is a measure of the mean annual global Normalized Difference Vegetation Index (NDVI), the most common measurement of the density of plant growth obtained by the EDIT Geoplatform [62]. NDVI is derived from satellite images over the entire globe. We also tested the effect of seasonality using precipitation seasonality (measured as the coefficient of variation of monthly means precipitation in mm) as predictor variable. These two variables explained a significant proportion of variance in a Principal Component Analysis (PCA) analysing a range of climatic and environmental variables within the distribution ranges of *Stegodyphus* (Additional file 1: Table S2). The two variables, vegetation productivity and precipitation seasonality, were only weakly correlated (Pearson’s r = −0.254).

### Statistical methods

First we applied Wilcoxon sum tests to compare differences in vegetation productivity (GVI) and precipitation seasonality among social and solitary species habitats.

To test the underlying hypotheses further, we ran logistic regressions on the *Stegodyphus* occurrences [63] with the presence (1) or absence (0) of social species as response variable, i.e., assessing the influence of the two predictor variables on the probability that any given *Stegodyphus* occurrence belongs to a social species. The three social species might show ecological differences. To account for these we defined three “social-species regions” based on the ranges of the three social species (Figure 1; Additional file 1: Table S1). Each solitary species record was assigned to the region of the social species with the nearest record. The regions were recoded as two binary dummy variables (Region 1, bin1 = 1, bin2 = 0; Region 2, bin1 = 0, bin2 = 1; Region 3, bin1 = 0, bin2 = 0). These region variables were not only used as main effects in the models, but also in region × environment terms to represent potential differences in the social species’ relationships to the two environmental predictors. Two interaction terms, among each of the two environmental effects and each of the three social-species regions (region 1, 2 or 3 in Figure 1; Additional file 1: Table S1) were computed to capture potential ecological differences among species across social-species regions [64]. The interaction terms were derived as the sum of binaries, each multiplied by the environmental variable (bin1* X_i_ + bin2* X_i_) (Figure 1).

Spatial autocorrelation is frequent in geographic data and will inflate significance levels if not properly handled, and may also lead to other statistical problems [64,65]. However, a number of methods exist to account for spatial autocorrelation. Here, we used the spatial eigenvector filtering approach [66], as it can readily be applied to any regression approach including logistic regression. Spatial filters are constructed based on a geographic distance matrix, and subsequent filters are orthogonal variables which describe space at increasingly fine spatial scales [67]. The first thirteen filters removed most of the spatial autocorrelation in the residuals of the models, with Moran’s I values being reduced to approximately zero, also in higher distance classes. To avoid excess of predictors, we chose six of the first thirteen filters, selected based on how well they captured geometry of the area and the amount of the spatial autocorrelation removed in the residuals [66]. One of the filters was excluded, as it correlated with vegetation productivity (sf4: Spearman’s r = 0.561, p = <.001); all the other filters did not correlate with vegetation productivity nor precipitation seasonality.

In order to test the hypothesized environmental drivers of social *Stegodyphus* occurrence, 10 logistic regression models (Table 1) were built in SAM v. 4.0 using various combinations of the total set of predictor variables. These comprised vegetation productivity, precipitation seasonality, the two region dummy variables, interaction terms between the two environmental variables and region, and six spatial filters. Explanatory power of the models was estimated using R^2^ adjusted by the maximum achievable R^2^ for the data [68] and the true skill statistic (TSS), also known as the Hanssen-Kuipers discriminant [69]. As a measure of the accuracy of presence-absence predictions, TSS is related to the kappa statistic, but avoids the latter’s dependence on prevalence [69]. Similar to kappa, TSS ranges from −1 to +1, with +1 indicating perfect agreement and values ≤0 indicating a predictive accuracy no better than random or worse [69]. Following [70], a nuanced verbal interpretation may be given as poor (<0.00), slight (0.00-0.20), fair (0.21-0.40), moderate (0.41-0.60), substantial (0.61-0.80), and almost perfect (0.81-1.00) (see e.g., [71]).

We used the Akaike Information Criterion for model selection [72], to estimate the relative magnitude and sign of the effects of productivity and seasonality on the occurrence of social and solitary spiders. Based on the number of parameters (K) and the log likelihood of each model, the AIC score of it can be used to compute AIC differences, ∆ AIC = AIC(*i*) - AIC(min), which provides information on relative support for each model *i*. Subsequently, Akaike weights (w_*i*_) are derived from ∆AIC, and show the strength of evidence for model support.

Akaike weights were calculated for each model and used to compute model-averaged regression coefficients (**β**_**A**_**)**[73]. From **β**_**A**_ we derived relative coefficient weights for vegetation productivity and precipitation seasonality across all models (as our variables of interest). Finally, odds ratios (**OR**_**A**_) for those two variables and their interaction terms were calculated from the model-averaged coefficients [72,73]. These odds ratios indicate the multi-model predicted change in odds of presence to absence for a unit change in the standardised predictor variable [63]. In this case, the odds were the odds that a given *Stegodyphus* occurrence belonged to a social species [74].

### Insect biomass in relation to habitat productivity and precipitation seasonality

To test the functional basis for interpreting the social/solitary occurrence-environment relationships, we assessed how prey availability (insect biomass) is related to vegetation productivity and precipitation seasonality across the distribution of *Stegodyphus*. We used a set of studies on insect seasonality and animal diets (of birds and spiders) which provided a measure of relative insect biomass across our study region. We did a search on Web of Science and Google Scholar using the terms ‘insect’, ‘arthropod’, ’prey’, ‘biomass’, ‘abundance’, ‘productivity’, ‘Africa’, ‘India’; including the references within papers citing the search results. In total 16 studies with site-specific insect biomass estimates were included in the analysis (Additional file 1: Table S3). The study areas included Africa, Middle East and Western Ghats of India. 12 out of the 16 studies were performed on aerial insects collected by sweeping, vacuum suckers, window traps, sticky or light traps. For details see references within Additional file 1: Table S3. Four studies were restricted to ground dwelling insects such as beetles and termites; however in particular termites but also beetles constitute a major component of the diet of social *Stegodyphus* (M. Majer & C. Holm, *pers. obs*, quantitative study of social spider diets in two *Stegodyphus* species). Therefore, the analysis of the correlation between insect biomass and habitat productivity encompasses relevant measures of insect biomass that represent potential prey of *Stegodyphus* spiders.

Furthermore, we pooled estimates of average monthly insect biomass for the period November-January from a subset of the previously selected studies to represent insect biomass during the breeding season of *Stegodyphus*, when prey demands should be highest [22]. For the later, we also used insect biomass estimates from six of our own field sites (three in Namibia; two in Israel; one in India) (M. Majer & T. Bilde, *unpubl. data*). The studies were done as parts of C. Holm’s MSc thesis and M. Majer’s PhD thesis at Aarhus University. Field work was done with permissions at Farm Hüttenhoff and Farm Uisib in Namibia, in collaboration with The Jacob Blaustein Institutes for Desert Research in Israel, and with permission at Kuppam Campus of Agastya International Foundation in India. The localities of the above studies were georeferenced, and vegetation productivity and precipitation seasonality values were extracted for these localities. We used linear mixed-effects models on biomass against vegetation productivity and precipitation seasonality as explanatory variables (vegetation productivity, precipitation seasonality, season and insect taxon as fixed; and sampling effort as a random effect), and then performed ANOVA analysis on the fixed effects. Insect biomass was transformed logarithmically to satisfy the assumption of normal distribution of residuals.

The logistic regression modelling was done in SAM 4.0 [75], while for the analysis of insect biomass the nlme package for linear-mixed models effects was used in R 2.13.2 [76]. All figures were also made in R 2.13.2.

## Competing interests

The authors have declared that no competing interests exist.

## Authors’ contributions

MM carried out the analyses and drafted the manuscript. MM, JCS and TB designed the study. JCS and TB helped draft the manuscript. All the authors read and approved the final manuscript.

## Supplementary Material

Additional file 1: Table S1. List of 17 species with their distribution ranges, level of sociality and numbers of records used in the analysis. **Table S2.** Principal components scores and loadings on the *Stegodyphus* presence matrix with environmental variables listed. **Table S3.** References for site-specific biomass estimates of insects used for our supplementary insect biomass analysis. **Figure S1.** Inserts of the species maps in the South African region of the Figure 1, where the spider distribution records are very dense. **Figure S2.** Boxplots of vegetation productivity and precipitation seasonality for occurrences of social and solitary *Stegodyphus* species in each of the three regions. **Figure S3.** Correlograms of Moran’s I on distance classes of the model residuals (models in Table 1). (DOCX 430 kb) Click here for file
