# Supplementary material for: Habitat productivity constrains the distribution of social spiders across continents – case study of the genus Stegodyphus
Source: Front Zool. 2013 Feb 23;10:9. doi: 10.1186/1742-9994-10-9 (PMC3599804; doi:10.1186/1742-9994-10-9)
Supplement: Additional file 1: Table S1. — List of 17 species with their distribution ranges, level of sociality and numbers of records used in the analysis. Table S2. Principal components scores and loadings on the Stegodyphus presence matrix with environmental variables listed. Table S3. References for site-specific biomass estimates of insects used for our supplementary insect biomass analysis. Figure S1. Inserts of the species maps in the South African region of the Figure 1, where the spider distribution records are very dense. Figure S2. Boxplots of vegetation productivity and precipitation seasonality for occurrences of social and solitary Stegodyphus species in each of the three regions. Figure S3. Correlograms of Moran’s I on distance classes of the model residuals (models in Table 1). (DOCX 430 kb) [file 1742-9994-10-9-S1.docx]

**Appendix S1.**

**Table S1.** List of 16 species with their distribution ranges, level of sociality and numbers of records used in the analysis.

| Species | Life-history | Distribution | Number of records in the dataset |
| --- | --- | --- | --- |
| *Stegodyphus africanus* | Solitary | Africa | 27 |
| *Stegodyphus bicolor* | Solitary | Southern Africa | 15 |
| *Stegodyphus dufouri* | Solitary | North, West Africa | 12 |
| *Stegodyphus dumicola* | **Social** | **Central, South Africa** | **99** |
| *Stegodyphus hildebrandti* | Solitary | Central, East Africa, Zanzibar | 6 |
| *Stegodyphus lineatus* | Solitary | Europe to Tajikistan | 62 |
| *Stegodyphus lineifrons* | Solitary | East Africa | 2 |
| *Stegodyphus manicatus* | Solitary | North, West Africa | 8 |
| *Stegodyphus mimosarum* | **Social** | **Africa, Madagaskar** | **66** |
| *Stegodyphus mirandus* | Solitary | India | 4 |
| *Stegodyphus nathistmus* | Solitary | Morocco to Aden | 4 |
| *Stegodyphus pacificus* | Solitary | Jordan, Iran, Pakistan, India | 11 |
| *Stegodyphus sabulosus* | Solitary | East, Southern Africa | 7 |
| *Stegodyphus sarasinorum* | **Social** | **India, Sri Lanka, Nepal** | **28** |
| *Stegodyphus tentoriicola* | Solitary | South Africa | 6 |
| *Stegodyphus tibialis* | Solitary | India, Myanmar, Thailand, China | 9 |
| TOTAL  Solitary (13 sp.) |  |  | 173 |
| Social (3 sp.) |  |  | **193** |

|  |  |  |  |  |
| --- | --- | --- | --- | --- |

*S. hisarensis and S. simplicifrons* were excluded due to the old, poor or no locality records. The only record for *S. tingelin* could not be georeferenced due to poor locality record. *S. annulipes and S. manaus* were excluded since their recorded occurrences are from Brazil, and the permanent sociality of *S. manaus* was described based on the observations of a few (juvenile and subadult) individuals.

Environmental data

Several climatic variables were obtained from the WorldClim dataset (monthly data from 1950-2000; Hijmans RJ, Cameron SE, Parra JL, Jones PG and Jarvis A [1] ). WorldClim calculates the annual/quarter means of several climatic variables by deriving them from monthly temperature and rainfall values measured around the world in the period 1960-2000. Seasonality is calculated by standard deviation (temperature, in °C * 10) or coefficient of variation (precipitation in mm).

In addition, habitat productivity and aridity indeces (Supplement 1) were used. As a proxy for habitat productivity, we used GVI, which is a measure of the mean annual global Normalized Difference Vegetation Index (NDVI), the most common measurement of the density of plant growth (obtained by the EDIT Geoplatform [2]. NDVI is derived from satellite images over the entire globe in a 18 year period (1982-2000). Original NDVI real values (from -1 to +1) were rescaled to a range from 1 to 255 (byte format). A yearly average (GVI) was computed for both mean and std NDVI by averaging the monthly means using the cell statistic function in Spatial Analyst setting cell size and extent to one of the monthly layers. An aridity index was obtained from the Global Aridity and PET Database (SCI, http://www.cgiar-csi.org/) [3].

For each presence locality, the corresponding environmental data were extracted in ArcGIS 9.3.1 (ESRI [4] from 19 environmental layers (table S2 in the supplementary material with more details on calculation of each variable), all resampled to 30’’ resolution (approx. 1-km² at the Equator).

**Table S2.** Principal components scores and loadings on the *Stegodyphus* presence matrix with environmental variables listed. Scores of first two principal components were 35 and 19%. Variable loadings were considered for the first three principal components, in order to choose the variables to be included in the model. The highlighted scores (in bold) of predictors were considered for the further logistic regression analysis; for the selection see Methods section. Climatic variables on annual and monthly temperature values, were computed in the same way; therefore some of them are also highly correlated (the same argument applies to the precipitation variables). These climate variables might have than scored high in the PCA analysis due to the spatial structure in the data, as climate is recognised to be the global driver of biodiversity patterns [5]. On the second axis precipitation seasonality had a high score, and was selected to build the models based on our precipitation seasonality hypotheses (see Introduction section).

| Environmental predictor | PCA1 | PCA2 | PCA3 |
| --- | --- | --- | --- |
| % Total variance explained | 39.12% | 20.47% | 11.30% |
| Precipitation Seasonality | -0.121 | 0.308 | 0.217 |
| GVI* | 0.272 | -0.071 | 0.249 |
| Annual mean temperature | 0.027 | 0.457 | -0.263 |
| Mean Diurnal temperature Range | -0.206 | 0.016 | 0.448 |
| Max Temperature of Warmest Month | -0.012 | 0.119 | 0.205 |
| Annual Temperature Range | -0.287 | -0.079 | 0.259 |
| Mean Temperature of Wettest Quarter | 0.069 | 0.390 | 0.026 |
| Mean Temperature of Warmest Quarter | -0.117 | 0.368 | -0.285 |
| Mean Temperature of Coldest Quarter | 0.146 | 0.412 | -0.224 |
| sqrt (Isothermality) | -0.253 | -0.134 | -0.078 |
| Sqrt (Annual mean Precipitation) | 0.343 | 0.029 | 0.133 |
| Sqrt (Precipitation of Wettest Month) | 0.303 | 0.138 | 0.187 |
| Sqrt (Precipitation of Driest Month) | 0.253 | -0.202 | -0.169 |
| Sqrt (Precipitation of Wettest Quarter) | 0.313 | 0.118 | 0.180 |
| Sqrt (Precipitation of Driest Quarter) | 0.274 | -0.178 | -0.166 |
| Sqrt (Precipitation of Warmest Quarter) | 0.293 | 0.035 | 0.254 |
| Sqrt (aridity**) | 0.342 | -0.032 | 0.090 |
| Log (Mean Temperature of Driest Quarter) | -0.039 | 0.242 | 0.270 |
| Log (Precipitation of Coldest Month) | 0.176 | -0.170 | -0.296 |

* GVI is a yearly average computed on the mean monthly NDVI values obtained from satellite imagery.

** Aridity Index values, as mean annual aridity was calculated as the ratio of annual precipitation over annual potential evapotranspiration (dimensionless unit), increase for more humid conditions, and decrease with more arid conditions.

**Table S3.** References for site-specific biomass estimates of insects used for our supplementary insect biomass analysis.

|  | Source | Location | Insect taxon | Period/  season |
| --- | --- | --- | --- | --- |
| 1 | Sinclair 1978 | Seronera; Serengeti | All insects | ann |
| 2 | Dingle& Khamala 1972 | Athi plains; Nairobi; Kenya | All insects | ann |
| 3 | Lack 1986 | Tsavo East National Park, Kenya | All insects | ann |
| 4 | Jetz 2003 | Comoe´ National Park, Ivory Coast | Aerial insects | ann |
| 5 | Murali 1993 | Mudumulai Sanctuary, Tamilnadu India | Aerial insects (arboreal) | ann |
| 6 | Pringle 2010 | Mpala Research Centre; Kenya | All aerial + arboreal arthropods | ann |
| 7 | Rautenbach 1988 | Luvuvhu river, Kruger National Park SRA | All aerial + arboreal arthropods | ann |
| 8 | Eggleton 2000 | Mbalmayo Forest Reserve, S Cameroon | Termites | ann |
| 9 | Davis 1996 | Gauteng Province Pretoria; SRA | Coleoptera (dung beetles) | ann |
| 10 | Krasnov 1996 | Negev; Israel | Coleoptera (Tenebrionidae) | ann |
| 11 | Schletwein 1984 | Karoo, N Cape RSA | All insects | ann |
| 12 | Riechert 1985 | M'Passa forest, Gabon | All insects | seas |
| 13 | Vohland 2004 | Gellap-Ost and Nabaos; Namibia | Coleoptera (Tenebrionidae and Scarabeidae) | seas |
| *1** | *Sinclair 1978* | Seronera; Serengeti | All insects | seas |
| *3** | *Lack 1986* | Tsavo East National Park, Kenya | All insects | seas |

*** These two studies were also used for the the estimates of seasonal insect biomass, since the trapping was done over severall months, including the period we were interested in (see Methods section).


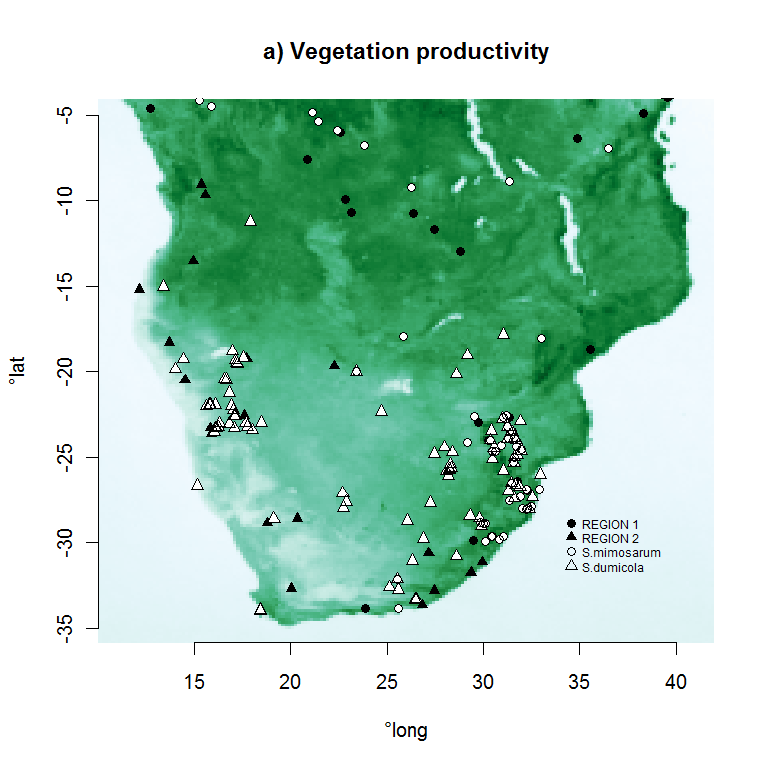

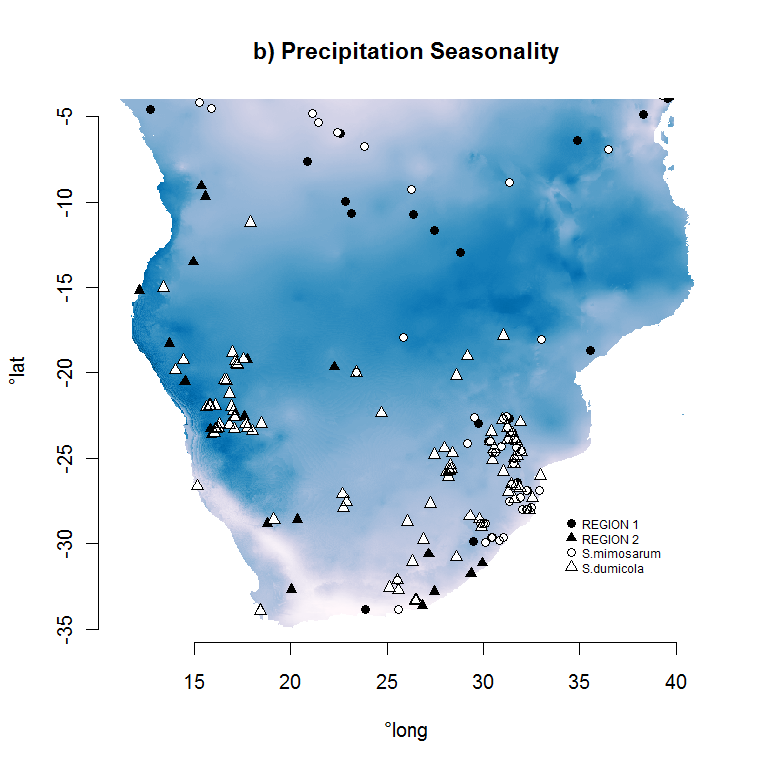


**Figure S1:** Inserts of the species maps in the South African region of Figure 1 (main text file), where the spider distribution records are very dense. Gradients of GVI (a) and annual precipitation seasonality (b) across the study area are shown in the same colours as in Figure 1. Two regions, defined to separate the distributions of the social species, are indicated by empty circles (region 1), and triangles (region 2). Empty and filled symbols indicate the occurrences of social and solitary species, respectively. The darker the green in (a), the more productive the continental area is. Likewise, the bluer the continental area in (b), the more seasonal it is in precipitation patterns.


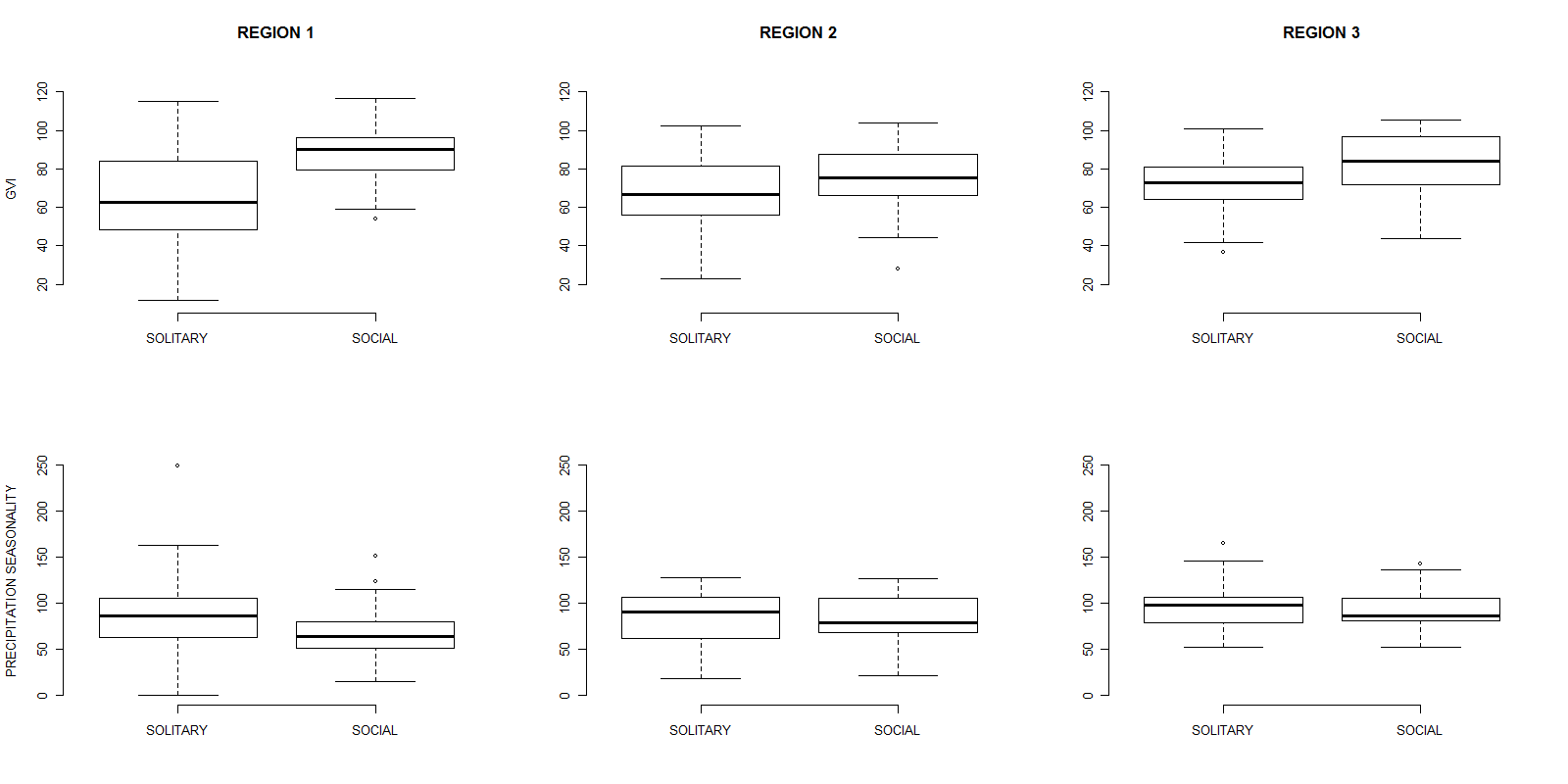


**Figure S2:** Boxplots of (a) vegetation productivity and (b) precipitation seasonality for occurrences of social and solitary *Stegodyphus* species (n = 193 and 173, respectively) in each of the three regions (defined in the Methods section, see maps in Figure 1 in the main text file). The extremes, the inter-quartile range, and the median are shown.


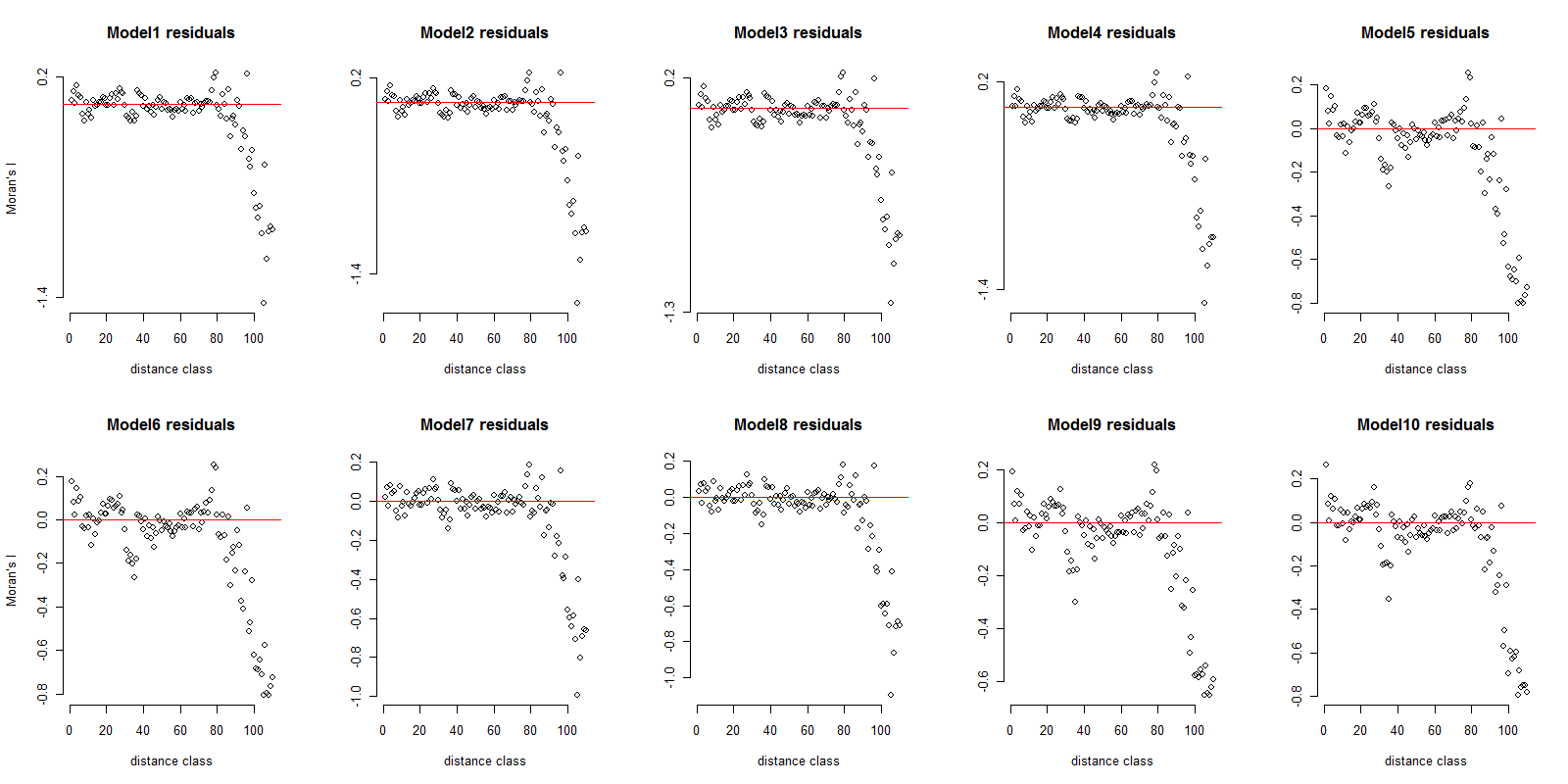


**Figure S3:** Correlograms of Moran’s I on distance classes of the model residuals (Table 1 in the main file lists the explanatory variables of each model). The most supported models according to the AIC criterion are: m1, m2, m3, m7 and m9 (table 1 in the main text file).Values of the Moran's I are very low in the largest distance classes, which is not unusual, as the sample size is low across most distant records.

**References**

1. Hijmans RJ, Cameron SE, Parra JL, Jones PG, Jarvis A: **Very high resolution interpolated climate surfaces for global land areas.** *International Journal of Climatology* 2005, **25:**1965-1978.

2. Lobo JM: **EDIT Geoplatform.** In *Book EDIT Geoplatform* (Editor ed.^eds.). City; 2007.

3. Zomer RJ, Trabucco A, Bossio DA, Verchot LV: **Climate change mitigation: A spatial analysis of global land suitability for clean development mechanism afforestation and reforestation.** *Agriculture, Ecosystems &amp; Environment* 2008, **126:**67-80.

4. (ESRI) ESRI: **ArcMap 9.3.** In *Book ArcMap 9.3* (Editor ed.^eds.), 9.3.1 edition. pp. Geographic information system (GIS) software. City: ESRI, Redlands, California; 2010:Geographic information system (GIS) software.

5. Pearson RG, Dawson TP: **Predicting the impacts of climate change on the distribution of species: are bioclimate envelope models useful?** *Global Ecology and Biogeography* 2003, **12:**361-371.
